# From motion to meaning: understanding students’ seating preferences in libraries through PIR-enabled machine learning and explainable AI

**DOI:** 10.3389/fpsyg.2025.1642381

**Published:** 2025-07-11

**Authors:** Gizem Izmir Tunahan, Goksu Tuysuzoglu, Hector Altamirano

**Affiliations:** 1 Department of Architecture, Dokuz Eylul University, Izmir, Türkiye; 2 Department of Computer Engineering, Dokuz Eylul University, Izmir, Türkiye; 3 UCL Institute for Environmental Design and Engineering, London, United Kingdom

**Keywords:** seat preference, occupancy monitoring, academic library, machine learning, explainable AI, spatial behavior, user comfort, PIR sensors

## Abstract

This study presents a comprehensive, data-driven investigation into students’ seating preferences within academic library environments, aiming to inform user-centered spatial design. Drawing on over 1.3 million ten-minute passive infrared (PIR) sensor observations collected throughout 2023 at the UCL Bartlett Library, we modeled seat-level occupancy using 24 spatial, environmental, and temporal features through advanced machine learning algorithms. Among the models tested, Categorical Boosting (CatBoost) demonstrated the highest predictive performance, achieving a classification accuracy of 72.5%, with interpretability enhanced through SHAP (Shapley Additive exPlanations) analysis. Findings reveal that seating behavior is shaped not by individual factors but by two dominant dimensions: (1) environmental controllability, including access to personal lighting and fresh air, and (2) distraction management, characterized by quiet surroundings, visual privacy, and low-stimulation workspace finishes. In contrast, features commonly presumed to be influential, such as desk width, fixed computer availability, or daylight alone, had minimal impact on seat choice. Despite extensive modeling and optimization, prediction accuracy plateaued at approximately 72%, reflecting the complexity and variability of human behavior in shared learning environments. By integrating long-term behavioral data with explainable machine learning, this study advances the evidence base for academic library design and offers actionable insights. These findings support design strategies that prioritize individual environmental control, as well as acoustic and visual privacy, offering actionable, evidence-based guidance for creating academic library environments that better support student comfort, focus, and engagement.

## 1 Introduction

Academic libraries play a pivotal role in supporting students’ learning, research, and well-being by offering spaces for intensive study and educational engagement. Among the many design aspects that shape their effectiveness, the configuration and quality of seating in academic libraries play a critical role in influencing students’ comfort, productivity, and well-being, especially for those who spend extended periods within these environments. Therefore, understanding what drives students’ seating preferences is essential to optimizing these spaces for their health, well-being, and academic achievements (Peng et al., 2022).

Seating design goes beyond mere furniture selection; it plays a direct role in shaping the learning experience by influencing students’ concentration, social interaction, and sustained cognitive effort. Ergonomically designed seating, tailored to users’ needs, helps mitigate discomfort associated with prolonged study sessions, supports healthy posture, and reduces physical strain, thereby enabling longer and more effective study periods (Applegate, 2009).

Numerous studies have highlighted that ergonomically designed seating reduces physical strain, enhances concentration, and supports cognitive engagement, whereas poor seating arrangements can hinder students’ ability to sustain attention and engage in long-duration study sessions (Uche and Okata Fanny, 2016).

The consequences of prolonged exposure to uncomfortable or poorly designed environments extend beyond discomfort, often leading to musculoskeletal problems such as back pain and poor posture (Bai et al., 2024) and even contributing to elevated stress levels (Merga, 2021). Furthermore, the broader physical environment, including spatial openness, proximity to resources, and the overall atmosphere, can influence students’ collaborative behaviors, communication styles, and engagement in academic tasks (Zheng et al., 2024). Seating preferences in libraries are not merely the result of students’ practical or habitual choices; instead, they reflect deeper connections to students’ physical comfort, psychological well-being, and social engagement (Applegate, 2009). Optimizing library seating is not only a matter of improving academic outcomes but also creating inclusive, comfortable, and health-supportive environments that respond to diverse users’ needs and promote their health and well-being.

Placing seating areas near essential resources, such as bookshelves, computers, and charging stations, enhances the overall usability of library spaces by allowing students to access necessary materials and technology easily (Khoo et al., 2016). In addition to functionality, the comfort and atmosphere of the seating environment play a significant role in reducing stress and promoting a positive study experience, which can increase students’ focus and willingness to engage in academic tasks (Bryant et al., 2009).

Offering a range of acoustical settings also enables students to choose study areas that match their individual concentration needs, thereby supporting more effective and sustained learning (Gordon-Hickey and Lemley, 2012). Libraries that provide various seating options are better positioned to support different study approaches, from quiet individual work to collaborative group activities. This adaptability helps accommodate students’ varying preferences and encourages peer interaction, which is essential for both academic success and collaborative learning (Montgomery and Miller, 2011). Comfortable and welcoming areas also promote social engagement, allowing students to connect with peers, form study groups, and build supportive academic networks (Shill and Tonner, 2004).

Providing seating arrangements that address both functional requirements and social preferences is essential for creating library environments that are inclusive, adaptable, and conducive to academic success. Responding to the diverse needs of students not only enhances comfort and reduces stress but also supports improved learning outcomes and fosters a stronger sense of community. Furthermore, understanding how students interact with the physical environment offers valuable insights into improving user satisfaction (Paone and Bacher, 2018) and optimizing spatial use and energy performance within the library (Fabi et al., 2012). Accordingly, designing seating environments that respond to student needs and expectations constitutes a strategic decision that enhances user experience and supports institutional objectives related to academic performance, space utilization, and operational efficiency (Bennett, 2011).

Comfort within a library is shaped by environmental and spatial factors, including lighting conditions, ambient noise, furniture design, spatial layout, and access to peers and resources (Uşma and Gürsoy, 2022). In academic library settings, seating preferences are shaped by a complex interaction between environmental conditions and individual user characteristics. Among environmental variables, the most consistently influential factors include natural lighting, access to outdoor views, ambient noise levels, spatial positioning relative to circulation zones, and furniture ergonomics (Izmir Tunahan and Altamirano, 2022e). The lighting environment and visual access to the outdoors, in particular, have been shown to affect users’ seat selections significantly. Multiple studies have documented users’ preference for seats adjacent to windows that provide both daylight and external views, as these features support mood enhancement and sustained cognitive performance (Keskin et al., 2017; Izmir Tunahan et al., 2022b; Gou et al., 2018).

Acoustic conditions likewise play a critical role. Users typically avoid noisier areas and favor quieter zones to minimize distraction and promote concentration (Manna and De Sarkar, 2024). The spatial proximity of seating to high-traffic locations, such as doors, corridors, or common pathways, can introduce both auditory and visual disturbances, making spatial positioning an important consideration in the selection process (Fan et al., 2022). Additionally, furniture characteristics, particularly their alignment with anthropometric standards, directly influence comfort and perceived usability (Osquei-Zadeh et al., 2012). Complementary design features such as seating layout, desk dividers, and task-specific lighting have also been associated with greater user engagement and are frequently mentioned as determinants in seat selection (Blair, 2023).

While these environmental variables are well-documented in the literature, users tend not to evaluate them equally; instead, individuals selectively attend to a limited set of salient cues during the decision-making process (Keskin, 2019). Such selective attention complicates efforts to establish direct correlations between specific environmental stimuli and seating behavior, as users are simultaneously exposed to multiple spatial and sensory inputs (Izmir Tunahan, 2022c). Consequently, the mechanisms underlying the interaction between occupants and their physical surroundings remain only partially understood, highlighting the need for further empirical investigation (Boyce, 2014).

Individual-level factors likewise play a critical role in seating behavior. Expectations regarding indoor environmental quality and behavioral tendencies vary across users and are often influenced by personality traits (Nel and Fourie, 2016) and the nature of the task at hand (Delzendeh et al., 2017). Prior experience and spatial familiarity also contribute to seat selection; frequent users may repeatedly choose the exact location based on favorable past experiences, whereas newcomers rely more heavily on perceivable cues such as lighting or sound conditions (Keskin, 2019). Psychological variables, including arousal, motivation, and anticipated performance outcomes, further shape cognitive strategies and spatial decisions (Boyce, 2014). Finally, seating availability imposes a practical constraint: early arrivals have greater flexibility and are thus more likely to secure their preferred location (Izmir Tunahan, 2022c).

Various methodological approaches have been employed to investigate individuals’ seating behaviors in library settings. These methods vary regarding realism, experimental control, and data richness, with studies broadly divided between laboratory-based experiments and real-world observations. Laboratory studies allow for controlled manipulation of the environmental variables but often lack ecological validity, as participants’ awareness of observation can alter their natural behavior. This limitation, coupled with the artificiality of laboratory contexts, restricts the generalizability of findings to real-life environments.

**Figure 1:**
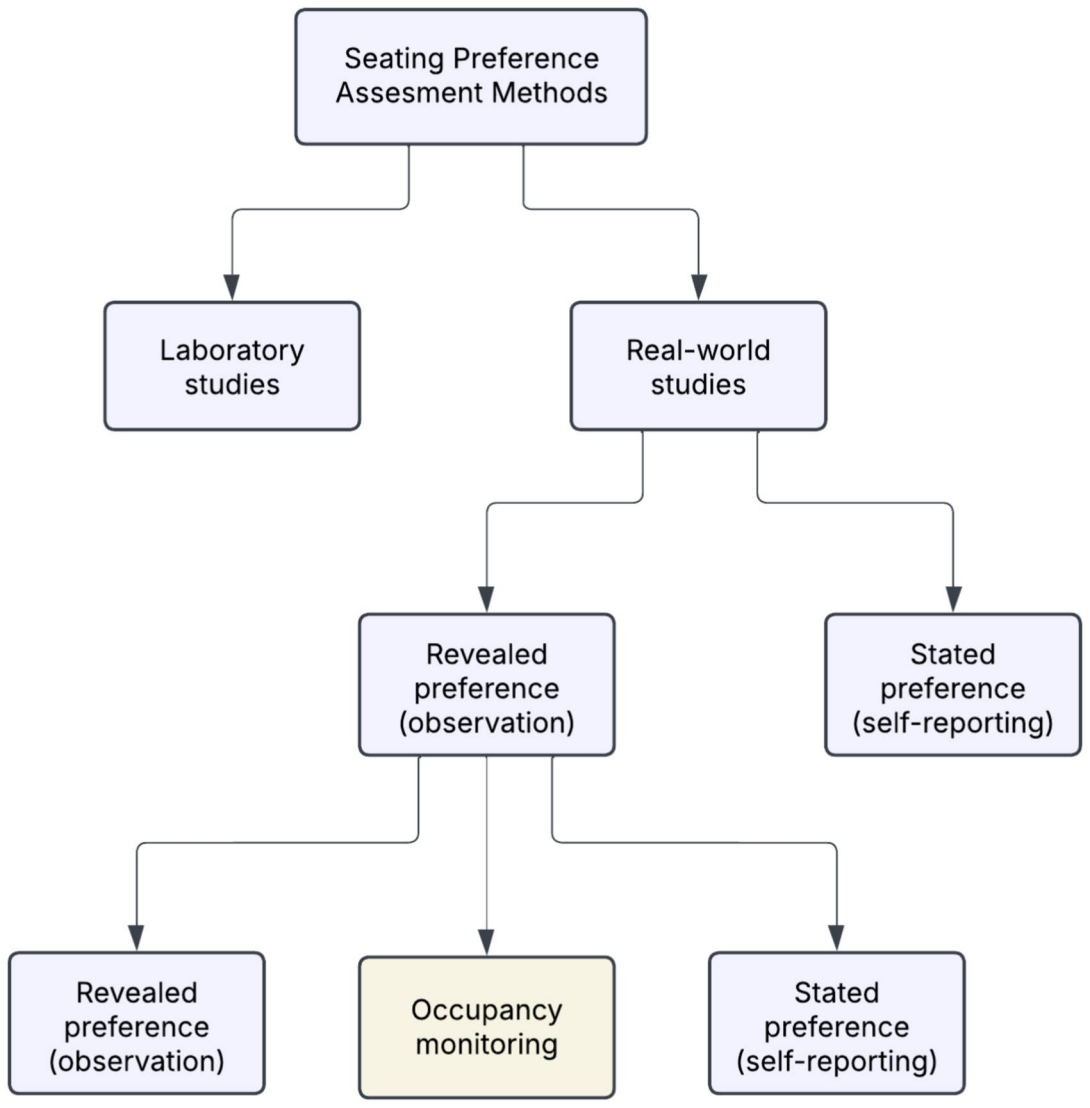
Overview of methodological approaches to assessing seating preferences in libraries (Izmir Tunahan, 2022c).

In contrast, field-based studies conducted in naturalistic settings offer the advantage of unobtrusive observation, minimizing behavioral bias so long as users are unaware of being monitored. While these approaches enhance ecological validity, they often provide less control over confounding variables (Keskin, 2019). In real-world settings, as illustrated in Figure 1, investigations of seating behavior typically adopt either revealed preference methods, which infer choice based on observed actions, or stated preference methods, which rely on self-reported perceptions, motivations, and intentions gathered through interviews or surveys (Izmir Tunahan, 2022c).

Revealed preference approaches offer robust data on actual behavioral patterns but often lack insight into the subjective reasoning behind seat selection. Conversely, stated preference methods provide valuable information on users’ preferences and expectations, yet are constrained by small sample sizes and limited generalizability. Accordingly, many scholars advocate for the combined use of both approaches to develop a more holistic understanding of seating behavior (Izmir Tunahan, 2022c).

Among revealed preference techniques, three commonly employed sub-methods are snapshot recordings, walk-through observations, and occupancy monitoring. Snapshot recordings capture periodic headcounts, such as every 30 min, to quantitatively track occupancy across different spatial zones. Walk-through observations provide richer qualitative data, such as noting movement paths and behavioral indicators like posture, activity type, or social grouping. However, due to their subjective nature and limited scale, such observations typically require multiple rounds to achieve generalizability. Finally, occupancy monitoring systems, often enabled by passive infrared (PIR) or motion sensors, facilitate large-scale, continuous, and non-intrusive tracking of seat usage. These systems offer high-resolution insights into spatial behavior at scale while preserving the ecological validity of the observed environment.

Occupancy monitoring refers to the systematic observation of the usage status of individual seats at specified time intervals (Izmir Tunahan, 2022c). This technique serves two primary purposes: quantifying spatial utilization for layout optimization (Min and Lee, 2020) and developing predictive models of occupant behavior using various sensor technologies (Liang et al., 2016). Occupancy monitoring plays a critical role in identifying the patterns that shape user behavior over time and space, particularly in studies examining human movement and spatial decision-making within built environments. Two main approaches are typically employed: manual headcounts and sensor-based systems. While commonly used, manual observation is labor-intensive and time-consuming, requiring considerable manual effort to collect seat-level data at consistent intervals. In contrast, sensor-based methods utilize advanced technologies such as PIR sensors, ultrasonic sensors, sound detectors, light-switch monitors, carbon dioxide sensors, and image-based tracking systems to detect and log seat occupancy continuously and autonomously (Pang et al., 2018).

A growing body of literature highlights the importance of designing flexible, student-centered library spaces that support concentration, encourage social interaction, and contribute to psychological comfort (Applegate, 2009; Khoo et al., 2016; Bryant et al., 2009; Gordon-Hickey and Lemley, 2012; Montgomery and Miller, 2011; Shill and Tonner, 2004). However, creating environments requires more than general design principles; it demands a detailed understanding of the factors influencing students’ seating decisions. Despite the increasing focus on user-centered library design, several critical gaps remain in the current literature. First, while numerous studies explore general environmental aspects, such as lighting, noise, or thermal comfort, only a few examine the specific characteristics of seating itself and how they relate to user satisfaction and preference (Izmir Tunahan, 2022c; Keskin et al., 2017; Izmir Tunahan et al., 2022b; Gou et al., 2018). Second, much of the existing research relies heavily on self-reported data, which can be subjective and susceptible to bias. Third, most investigations capture only short-term behaviors, lacking long-term, context-rich insights into how students actually use library seating over time.

As a result, there is a pressing need for objective, data-driven methodologies that can track behavioral patterns across extended periods and uncover subtle interactions between users and their environments (Izmir Tunahan, 2022c; Manna and De Sarkar, 2024; Fan et al., 2022; Osquei-Zadeh et al., 2012; Blair, 2023). Despite their capacity to generate high-resolution, long-term datasets, sensor-based approaches remain underutilized in seating preference research. This is primarily due to the high cost and logistical complexity of installing dedicated sensors on every seat or cluster of seats under observation (Izmir Tunahan, 2022c). As a result, many studies either rely on manual counting or limit sensor deployment to a select subset of seating areas, thereby constraining both the spatial and temporal resolution of the analysis (Chamilothori et al., 2016).

This study addresses these gaps by employing a novel, computational approach to investigate seating preferences in a real-world academic setting. Using passive infrared (PIR) motion sensors installed beneath each desk in the UCL Bartlett Library, a year-long dataset of occupancy records was collected and linked with detailed environmental and spatial variables, such as daylight exposure, access to fresh air, noise level, proximity to circulation routes, and desk configuration. Unlike earlier research relying on short-term observation windows or self-reported preferences, this study offers a comprehensive, objective dataset at the seat level.

Advanced machine learning (ML) models were applied to analyze this extensive dataset (over 1.3 million 10-min observations) and uncover the key factors influencing seat selection. These included both traditional classifiers, such as decision trees and logistic regression, and ensemble learning models, such as random forest, gradient boosting, Extreme Gradient Boosting (XGBoost), Light Gradient Boosting Machine (LightGBM), and Categorical Boosting (CatBoost). To further enhance model performance and interpretability, a mutual information-based feature selection process was implemented, followed by SHAP (Shapley Additive exPlanations) analysis to quantify the contribution of each feature to the model’s predictions.

Despite the emergence of PIR and real-time analytics in library management, few studies to date have explicitly identified which spatial and environmental factors most strongly influence seat selection, nor have they integrated state-of-the-art explainable machine learning techniques to generate design-oriented guidance. Addressing this gap, the present study couples a year-long dataset comprising over 1.35 million PIR sensor records with gradient boosting models and SHAP-based interpretability tools. This study offers a robust, multidimensional perspective on how students interact with library spaces by combining long-term behavioral monitoring with interpretable artificial intelligence techniques. The findings deepen our understanding of spatial behavior in academic settings and provide actionable insights for designing and managing library environments. In particular, the results reveal the most influential factors, such as lighting quality and spatial exposure, and those commonly assumed to be essential but shown to have limited impact, challenging prevailing assumptions in library design. This research has the potential to inform evidence-based strategies that enhance comfort, reduce stress, and support student well-being and academic performance through thoughtful and responsive spatial planning.

The previous studies highlighted that while numerous environmental, spatial, and individual factors are acknowledged to influence students’ seating choices in academic libraries, their precise interplay and relative importance, particularly when assessed through objective, long-term behavioral data, remain less clearly defined. Existing research often relies on short-term observations or subjective self-reports, and there is a noted underutilization of sensor-based data and advanced computational methods to uncover nuanced, context-rich insights into actual seat usage over extended periods. Furthermore, while machine learning offers potential for modeling such complex behaviors, the translation of these models into actionable, design-oriented guidance through explainable AI (XAI) techniques, particularly methods like SHAP analysis, is an emerging area in this domain.

This study, therefore, employs a novel, computational approach, leveraging a comprehensive year-long PIR sensor dataset from the UCL Bartlett Library and advanced machine learning techniques. This approach addresses the identified limitations by providing objective, granular data on seat-level occupancy and the contextual variables. Given this methodological framework and the identified knowledge gaps, several critical lines of inquiry emerge. Firstly, to build a foundational understanding from this rich dataset, it is essential to determine which specific attributes of the library environment demonstrably drive long-term seating patterns, a need underscored by the existing literature seeking to move beyond general principles to a detailed understanding of influencing factors. Secondly, with the application of sophisticated predictive models as outlined in this study’s methodology, it becomes imperative to evaluate their efficacy in capturing the complexities of seat selection behavior and to benchmark their performance. Finally, to ensure this research yields practical value for library design and management, it is crucial to move beyond predictive accuracy to uncover the underlying drivers of these predictions interpretably, specifically through SHAP analysis employed in this study, thereby facilitating evidence-based design decisions.

To guide this investigation, the following research questions are posed:

RQ1: Which spatial and environmental attributes most strongly influence long-term seat occupancy in an academic library?

RQ2: How accurately can advanced machine learning models predict seat utilization, and how do their performances compare to baseline classification algorithms?

RQ3: What design-relevant insights can be derived from explainable AI techniques (e.g., SHAP) to inform more user-responsive library seating layouts?

## 2 Materials and methods

### 2.1 Field site

**Figure 2:**
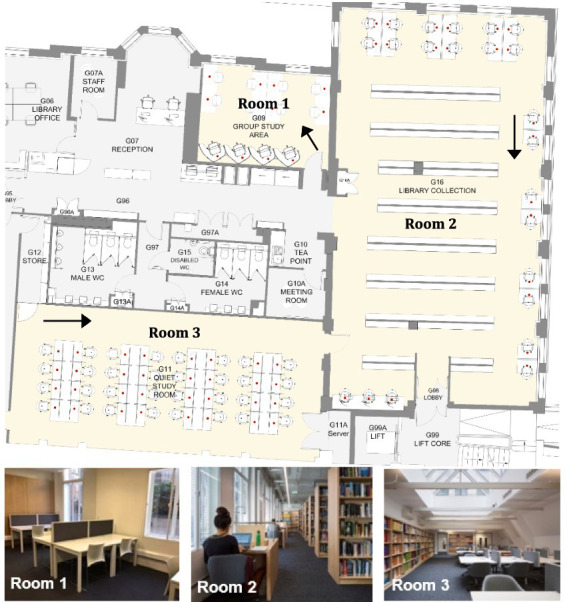
The floor plan of the UCL Bartlett Library [taken from Izmir Tunahan et al., 2022b]. Arrows show the viewpoints of accompanying photos.

This empirical study was conducted at the UCL Bartlett Library, located on the ground floor of the Central House in London, UK. The library comprises three distinct study rooms, each characterized by unique spatial and environmental features that make the site particularly well-suited for analyzing seat selection behavior. Room 1 includes two north-facing windows and features a combination of eight shared desks and four individual study cubicles. Room 2 is comparatively more open and better illuminated, with multiple windows facing north and east. It contains twelve shared desks and eleven individual desks. Room 3 is a large open-plan area lit primarily by two overhead skylights, accommodating 32 shared desks arranged in a single, flexible layout (Figure 2).

In addition to room-level differences, individual desks vary in surface color, divider height, chair type, and the presence or absence of reading lamps, among other design attributes (Izmir Tunahan and Altamirano, 2022d). These spatial and material variations provided a robust context for examining how environmental and furniture-related characteristics influence students’ seating preferences in a naturalistic academic setting.

### 2.2 Data collection

**Figure 3:**
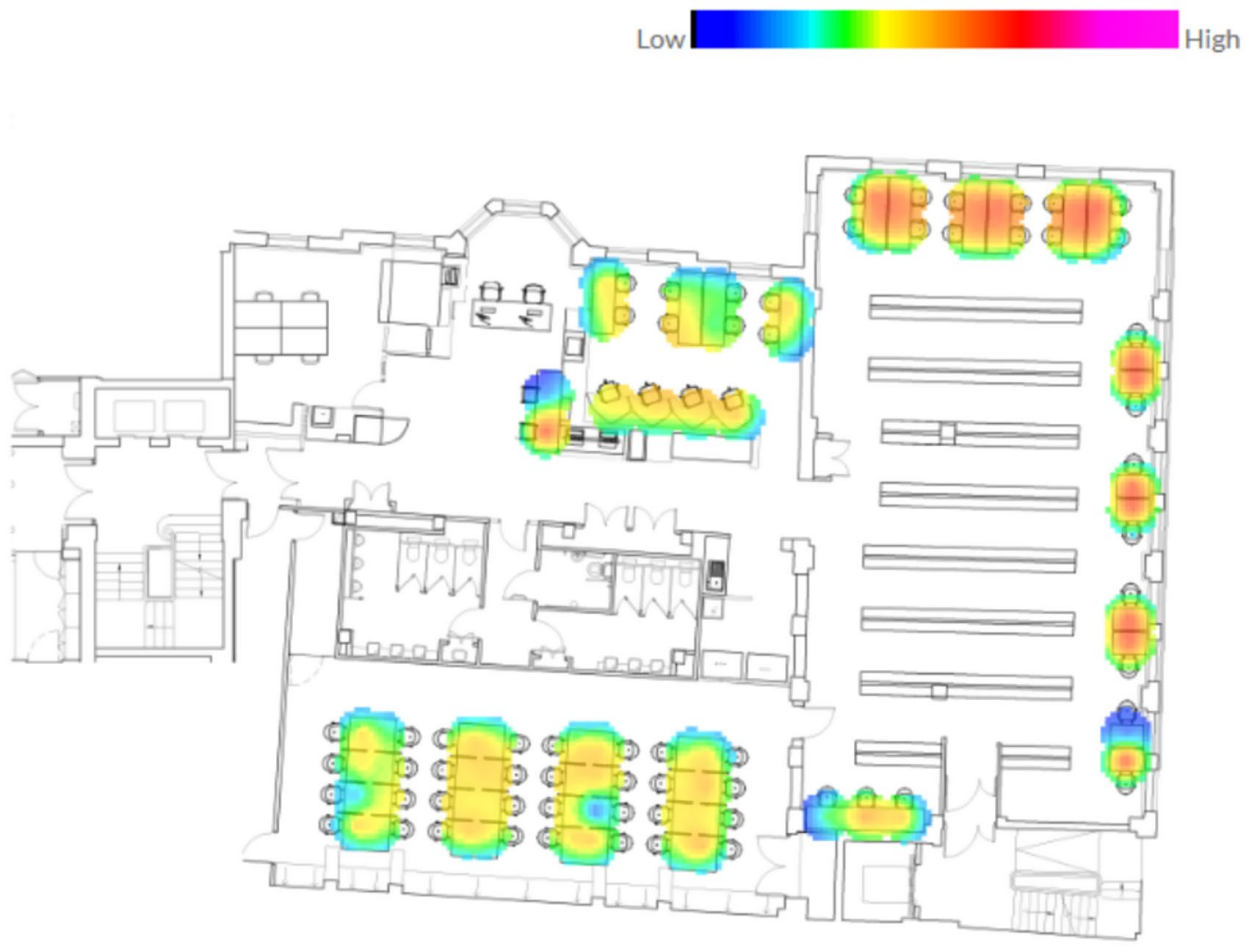
Heatmap of desk occupancy patterns throughout the study period (January–December 2023).

PIR sensors underneath each desk collected 10-min occupancy data for each desk in the Bartlett Library from January 2023 to January 2024, which was then retrieved from the OccupEye Cloud. Previously, the authors benefited from this database for various purposes: to understand the impact of daylight availability on library users’ seating preferences (Izmir Tunahan and Altamirano, 2022e) and to explore the changes in the use of study spaces before and after the pandemic (Izmir Tunahan and Altamirano, 2022d). Those studies, however, analyzed the average occupancy rates of desks and rooms on a daily, weekly, monthly, and annual basis rather than evaluating each desk individually, such as desks and rooms with more and less demand, desk preference order, and length of stay at the same desk. In those studies, a desk was considered occupied if a seat was occupied for at least 90% of a defined time period (Figure 3).

Since assessing each seat’s occupancy status in 10 min against changing daylight circumstances throughout the year was impossible, this enormous dataset could not be evaluated using SPSS or other statistical software. In contrast, this study utilizes machine learning algorithms to assess the occupancy condition of each seat in ten-minute intervals over 2023 against various desk parameters, as illustrated in Table 1.

**Table 1:** Input features, descriptions, and values for seat occupancy prediction models.

**Attribute name**	**Explanation**	**Values**
Date	Date on which the data was collected	{Year, Month, Day}
Time slot	Time of data collection (10-min intervals)	{Hour: Minute}
Daylight availability	Estimated daylight exposure at the desk, measured in lux, based on daylight factor calculations and external illuminance data	Integer value (relative range; higher indicates more daylight)
Desk divider type	Type and position of the desk divider	“Yes, in front of, short,” “Yes, in front of, long,” “Yes, surrounding, long,” “Yes, beside, short,” “No desk divider”
Sharing capacity	Number of users assigned per desk	{0, 1, 2}
Outdoor view quality	Level of visibility to outdoor or sky elements from the desk	“Partially outdoor view,” “Full outdoor view,” “Sky view,” “Building view,” “No outdoor view”
Reading lamp availability	Availability of a reading lamp at the desk	“Yes,” “No”
Access to fresh air	Accessibility to manually openable windows to get fresh air	“Yes,” “No”
Computer availability	Availability of a built-in computer on the desk	“Yes,” “No”
Desk width	Physical width category of the desk	“Narrow,” “Wide”
Divider color	Surface color of the desk	“White,” “Wood”
Desk divider color	Color or texture of the attached desk divider	“Dark color texture,” “Wood,” “Not applicable”
Desk-divider contrast	The degree of visual contrast between the desk and its divider	“Yes,” “No,” “Not applicable”
Quiet zone	Classification of the room as a silent or quiet area	“Yes,” “No”
Thermal exposure from window proximity	Exposure to thermal discomfort due to proximity to windows	“Yes,” “No”
Corner position	Placement of the desk in a corner of the line	“Yes,” “No,” “Not applicable”
Desk those faces, Wall	Orientation of the desk directly toward a wall	“Yes,” “No”
Circulation exposure level	Spatial and visual relationship of the desk to nearby circulation paths	“Seats that face away from a corridor (and thus movement flow) directly (1),” “Seats that have direct, straight-ahead visual contact with the main corridor (2),” “Seats that represent immediate side-by-side directness (3),” “Seats that have a distanced side-by-side directness with corridors (4)”
Proximity to entrance	Relative distance from the desk to the nearest room entrance	“Very close,” “Moderate,” “Far,” “Very far”
Visual openness score	Perceived spatial openness at the desk	An integer between 0 and 8 representing a value from “very narrow” to “very good”
Visual exposure index	The estimated number of users who can view the screen from nearby positions	Integer (0–12); higher values indicate greater visibility
Room type	Functional category of the room where the desk is located	“Small,” “Main,” “Hot desk space,” “Skylight”
Season	Seasonal period during which the data was collected	“Autumn,” “Winter,” “Spring,” “Summer”
Term status	Academic calendar phase at the time of collection	“Term time,” “Out-of-term time,” “Holiday”
Observed seat use	Occupancy status of the seat at the recorded time	“Yes,” “No”

The authors’ previous research on the link between daylight availability and library users’ seating behavior demonstrated that daylight availability strongly motivated students to choose specific seats with abundant daylight (Izmir Tunahan et al., 2022b; Izmir Tunahan and Altamirano, 2022e; Izmir Tunahan and Altamirano, 2022a). This study also aimed to validate their prior work with long-term occupancy data and previously used surveys, interviews, daylight boundary line drawings, and average desk occupancy data acquired from sensors. For this aim, the occupancy status of each desk in the library was recorded at 10-min intervals over a year and compared to the amount of daylight available on that desk at that time. The occupancy data was obtained from motion sensors in 10-min intervals, like in other parts of the study. Daylight availability at a specific location and time was determined through a combination of daylight simulations conducted at the Bartlett Library, as referenced in (Izmir Tunahan, 2022c), and external illuminance data collected at 10-min intervals in London from Public Health England for 2023. The instant internal illuminance for each seat was calculated using the external illuminance at the given time intervals and the daylight factor obtained from the simulations, where the daylight factor is defined as the ratio of indoor horizontal illuminance to outdoor daylight illuminance.

### 2.3 Dataset description

The dataset used in this study was derived from the UCL Bartlett Library, encompassing detailed seat-level occupancy data for 67 individual desks. Occupancy was recorded at 10-min intervals between January and December 2023, with each record indicating whether a given seat was occupied (“Yes”) or unoccupied (“No”). Data collection was conducted automatically during operational hours, from 9:00 a.m. to 7:50 p.m. daily, resulting in a total of 1,347,654 individual records. In addition to the binary occupancy status, the dataset includes 24 contextual and spatial attributes detailed in Table 1, capturing a wide range of environmental, temporal, and design-related factors. The dataset is approximately balanced, consisting of 741,977 “No” entries and 605,677 “Yes” entries. Importantly, the dataset is complete, with no missing values across recorded attributes.

To support temporal analysis, the original “Time” attribute was converted into a categorical variable comprising four distinct periods: morning (before 10:00 a.m.), noon (10:00 a.m. to 2:00 p.m.), afternoon (2:00 p.m. to 6:00 p.m.), and evening (after 6:00 p.m.). Season and Academic Calendar Status were additional features derived from the “Date” attribute. The Season variable classified each record into one of four meteorological categories: Spring (March–May), Summer (June–August), Autumn (September–November), or Winter (December–February). The Academic Calendar Status variable indicated whether each entry occurred during term time, out-of-term periods, or designated holiday closures, including bank holidays and reading weeks, based on the official UCL academic calendar. The original “Date” attribute was subsequently excluded from model training and analysis.

The complete list of features used in the analysis is presented in Table 1, along with corresponding definitions and value types. The Visual Openness Score was rated on a scale from 0 to 8, where lower values indicate greater enclosure (e.g., corner desks or those surrounded by dividers), and higher values represent more open, exposed locations (e.g., centrally located desks or skylit zones). Scores were derived through a combination of on-site assessments and spatial simulation metrics. The Visual Exposure Index quantifies the number of other users who can potentially view a desk’s screen based on line-of-sight proximity. Scores range from 0 (fully private) to 12 (maximum visibility) and were calculated through systematic visibility assessments across the library floor plan. Additionally, several variables include a “Not applicable” category to represent cases where the feature does not logically apply to a specific desk, such as Desk Divider Color without a divider or Corner Position for centrally located desks. These values were retained to preserve the integrity of categorical representations during model development.

### 2.4 Applied machine learning methods for seat occupancy prediction

This section outlines the machine learning algorithms utilized in the experimental studies. The entire implementation was carried out using Python in Google Colab. Two widely used traditional models, Decision Tree (DT) and Logistic Regression (LR), were first applied. A Decision Tree is a non-parametric supervised learning algorithm that splits the dataset into subsets based on feature values, forming a tree-like model of decisions (Breiman et al., 1984). It recursively partitions the feature space into regions associated with specific target outcomes, making it both intuitive and interpretable for classification tasks (Azam et al., 2023). Logistic Regression, on the other hand, is a linear classifier that predicts the probability of class membership using a logistic function (Hosmer et al., 2013; Zheng et al., 2024). It is computationally efficient and often serves as a strong baseline, especially when the relationship between features and the target is approximately linear.

To enhance predictive performance beyond what individual base models can achieve, this study employed five advanced ensemble learning methods: Random Forest (RF), Gradient Boosting Machine (GBM), Extreme Gradient Boosting (XGB), Light Gradient.

Boosting Machine (LGBM), and Categorical Boosting (CatBoost). Ensemble learning is a machine learning paradigm that combines the outputs of multiple models, often called “weak learners,” to produce a more accurate and robust prediction than any single model alone.

Random Forest enhances model robustness by aggregating the predictions of multiple decision trees trained on bootstrapped samples and random feature subsets, reducing the risk of overfitting (Breiman, 2001; Sun et al., 2024). GBM, a foundational ensemble method, builds models its predecessors (Friedman, 2001). XGBoost improves upon the traditional GBM approach by incorporating regularization techniques and supporting parallel computation, which makes it both accurate and scalable (Chen et al., 2016; Ma et al., 2021). LightGBM further optimizes training efficiency using histogram-based feature binning and a leaf-wise tree growth strategy, enabling it to effectively handle large-scale datasets with high dimensionality (Machado et al., 2019; Li et al., 2023). CatBoost is a gradient boosting framework particularly designed to handle categorical features without the need for extensive preprocessing (Prokhorenkova et al., 2018). Using techniques such as ordered boosting, CatBoost mitigates overfitting and provides reliable performance with minimal tuning. Its ability to natively process categorical variables and deliver high accuracy makes it exceptionally advantageous in real-world tabular data scenarios (Guo et al., 2023; Geeitha et al., 2024). Collectively, these models provide a well-rounded toolkit for modeling seat preference behavior, offering a balance between interpretability from simpler models and predictive strength from advanced ensemble techniques.

The models DT, LR, GBM, and RF were implemented using the scikit-learn (sklearn) library, while XGB, LGBM, and CatBoost were developed using their respective libraries: xgboost, lightgbm, and catboost. Model evaluation was conducted using 5-fold cross-validation, a robust validation strategy that partitions the dataset into five equal subsets. In this approach, each subset is used once as the test set while the remaining four subsets serve as the training data, ensuring that the model is assessed across multiple data splits to improve reliability and reduce variance in performance estimation.

## 3 Data analysis and results

**Figure 4:**
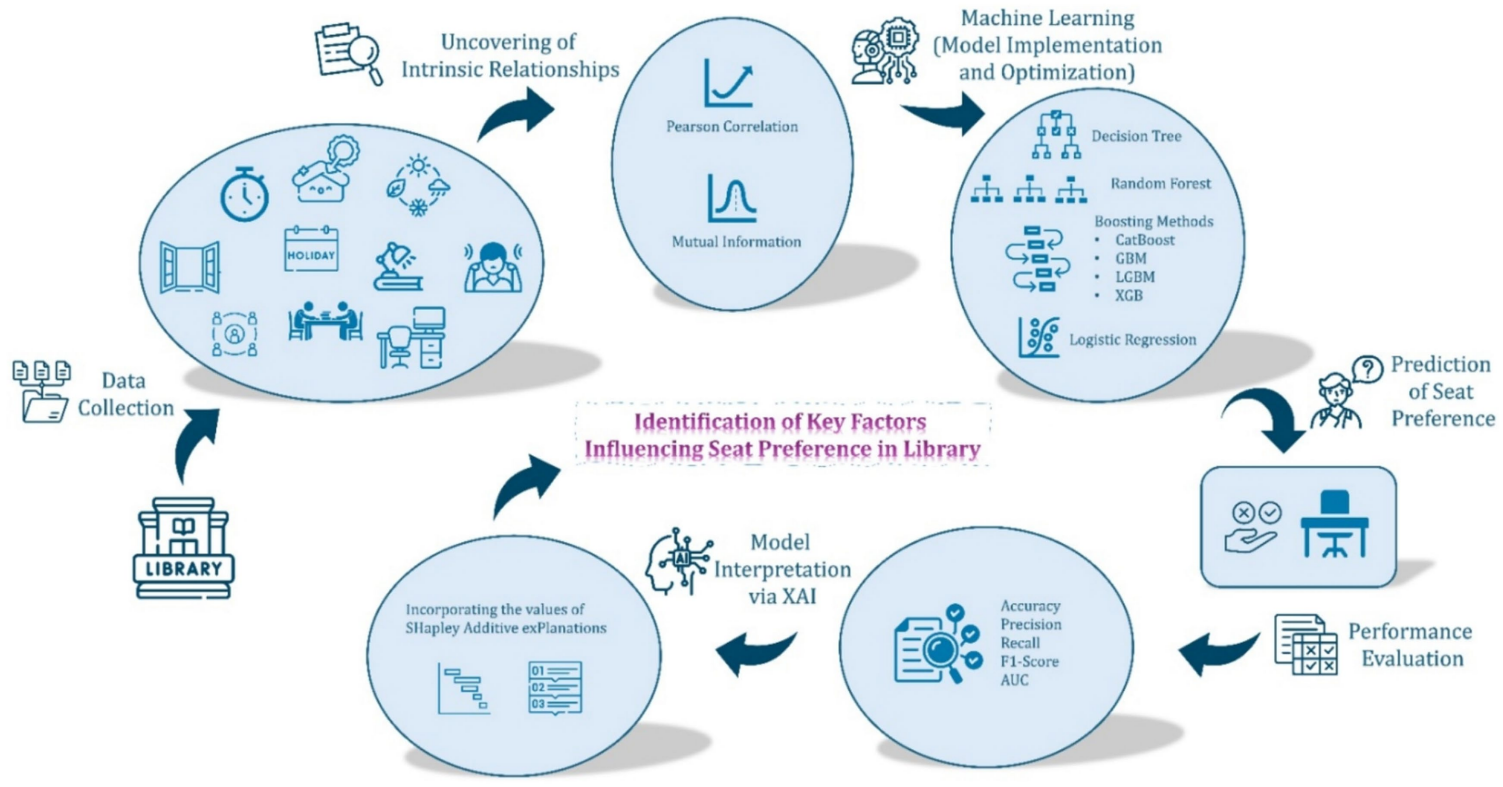
Methodological framework of the study.

This section presents the analytical findings based on over 1.3 million seat-level observations collected throughout 2023 at the UCL Bartlett Library. As seen in Figure 4, both linear (Pearson correlation) and nonlinear (Mutual Information) feature evaluation techniques were applied to identify the factors shaping student seating preferences, prior to training machine learning (ML) models. Seven classifiers were developed, optimized, and validated using 5-fold cross-validation. The best-performing model, CatBoost, was further interpreted using SHAP (Shapley Additive exPlanations) to provide transparent, model-agnostic insights into feature importance. The results are structured into six sequential stages: exploratory correlation analysis, MI-based feature selection, model implementation and tuning, performance evaluation, model comparison, and final interpretation via explainable AI.

### 3.1 Dataset overview

**Figure 5:**
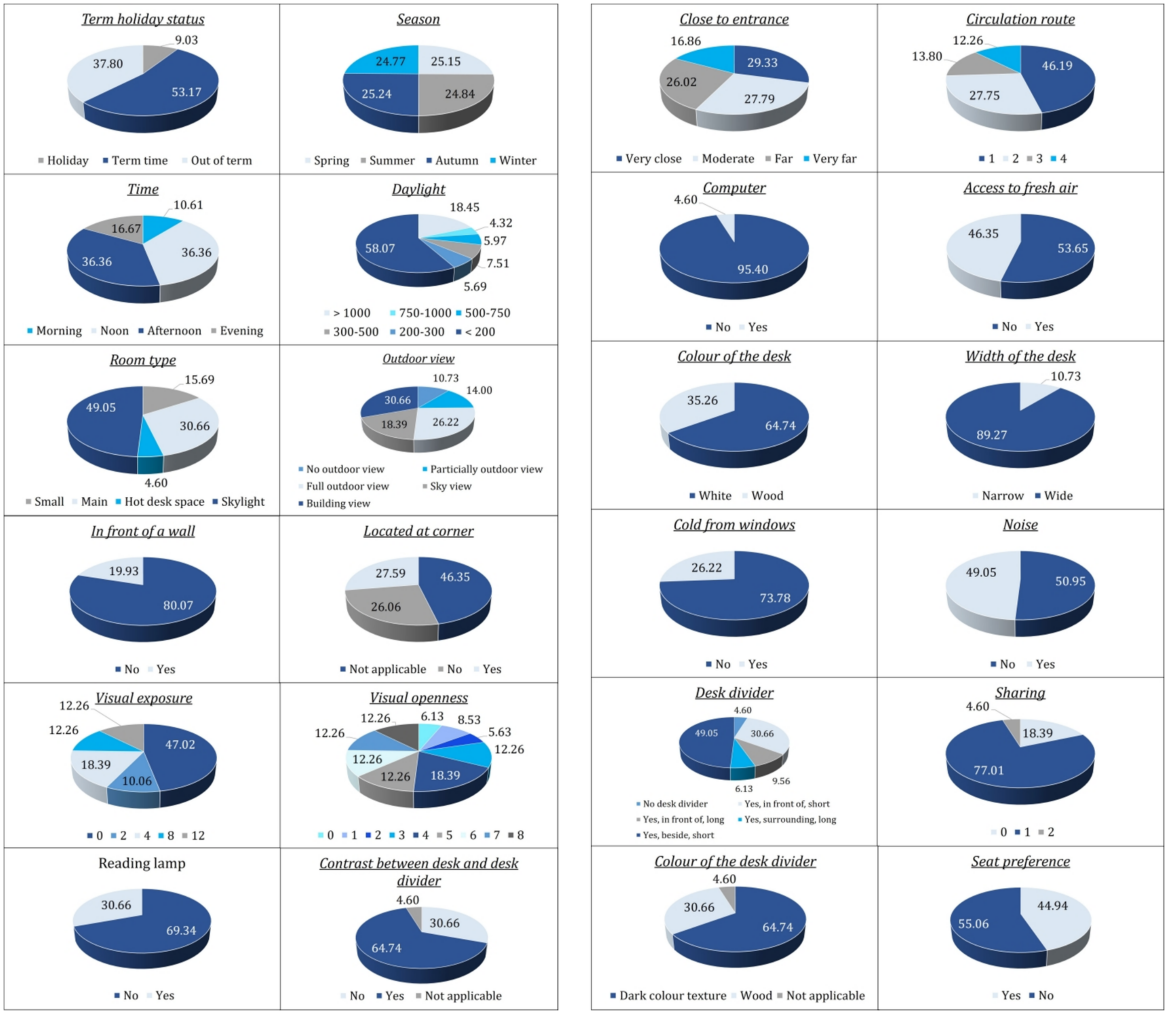
Distribution of dataset records across categories of key temporal, environmental, and spatial attributes.

**Figure 6:**
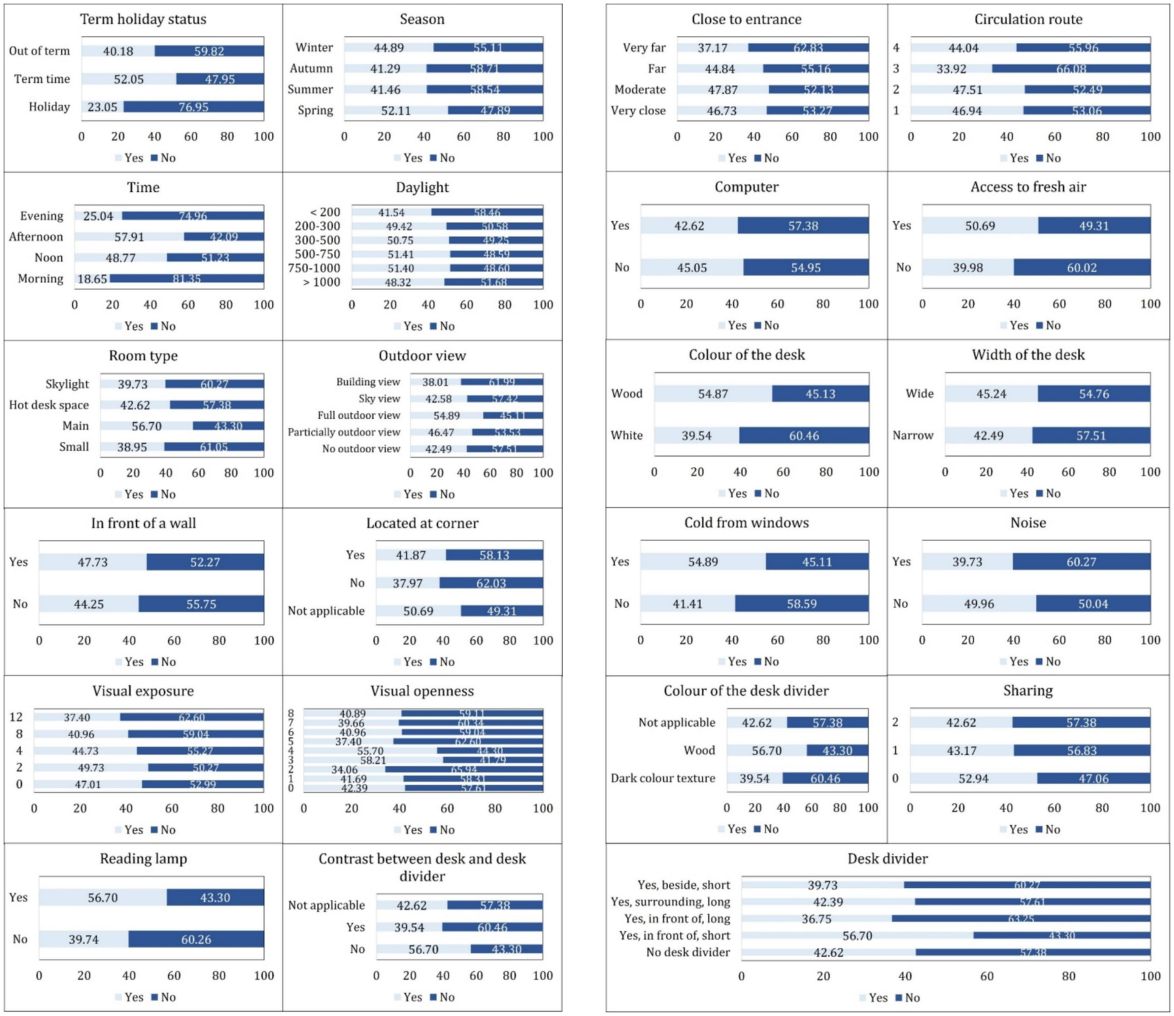
Observed seat occupancy proportions for categories of various environmental and spatial attributes.

Figures 5, 6 establish the following empirical context for the inferential analysis. It depicts the distribution of the 1.3 million 10-min passive-infrared (PIR) observations across all categorical variables and shows the proportion of observations in which each seat was recorded as occupied (“Yes”) or vacant (“No”). Temporal and spatial patterns emerge clearly in the data. The majority of observations were logged during term time (53%), followed by out-of-term periods (38%) and holiday periods (9%). Seat use was relatively balanced across Spring, Summer, Autumn, and Winter, although Spring exhibited the highest instantaneous occupancy (52%). The library was busiest in the Morning and at Noon; these two periods together yielded more than 70% of all “Yes” records, whereas the Evening period contributed only about 11%. Simulated daylight levels were predominantly low, with illuminance values under 500 lux comprising nearly two-thirds of the dataset. Around 70% of seats lacked an outdoor view, and 80% were not positioned directly in front of a wall. Approximately 25% were located at a corner, and visual openness and exposure metrics were centered on the mid-range, reflecting a balanced spatial composition. Only 30% of records indicated the presence of a reading lamp, and 35% showed a contrast between desk and divider finish.

Furniture and proximity attributes are also detailed. Seat locations were evenly distributed across entrance-distance and circulation-route bands, though nearly half fell on the innermost circulation loop. Fixed computers were available at only 4.6% of seats, suggesting that seats offered access to operable windows, and those with individual fresh-air control displayed visibly higher raw occupancy rates. Desk color was predominantly white (65%), and wide desks outnumbered narrow desks nearly tenfold.

Approximately one in four seats (26%) were positioned in locations potentially affected by thermal discomfort due to their proximity to windows, while the presence of ambient noise was evenly distributed across the dataset. Desk dividers were absent in roughly half the cases; when present, the most common form was a surrounding long panel. Visual contrast between desk and divider was absent in two-thirds of instances; dark textures, often thought to enhance privacy, were the least frequent and least occupied.

Conditional occupancy rates, also shown, reveal several influential predictors. Seats with reading lamps were occupied 57% of the time, compared to 40% for those without. The contrast between the desk and the divider was also underutilized.

### 3.2 RQ1: spatial and environmental predictors of seat occupancy

#### 3.2.1 Exploratory linear associations

As an initial step, the relationships between features were examined using Pearson correlation coefficients, which measure the strength and direction of linear relationships between pairs of variables (Lee Rodgers and Wander, 1988). The values range from −1 to 1, where values closer to ±1 indicate a stronger linear relationship, and values near 0 suggest little to no linear correlation.

The Pearson correlation coefficient, *r*, is calculated using Equation 1, in which $x_{i}$ and $y_{i}$ are individual data points, whereas x ® and y ® are the mean values of the n observations of the features X and Y, respectively.


$$r=\frac{\sum_{i=1}^{n}(x_{i}-\bar{x})(y_{i}-\bar{y})}{\sqrt{\sum_{i=1}^{n}{\left(x_{i}-\bar{x}\right)}^{2}}\sqrt{\sum_{i=1}^{n}{\left(y_{i}-\bar{y}\right)}^{2}}}$$ (1)

**Figure 7:**
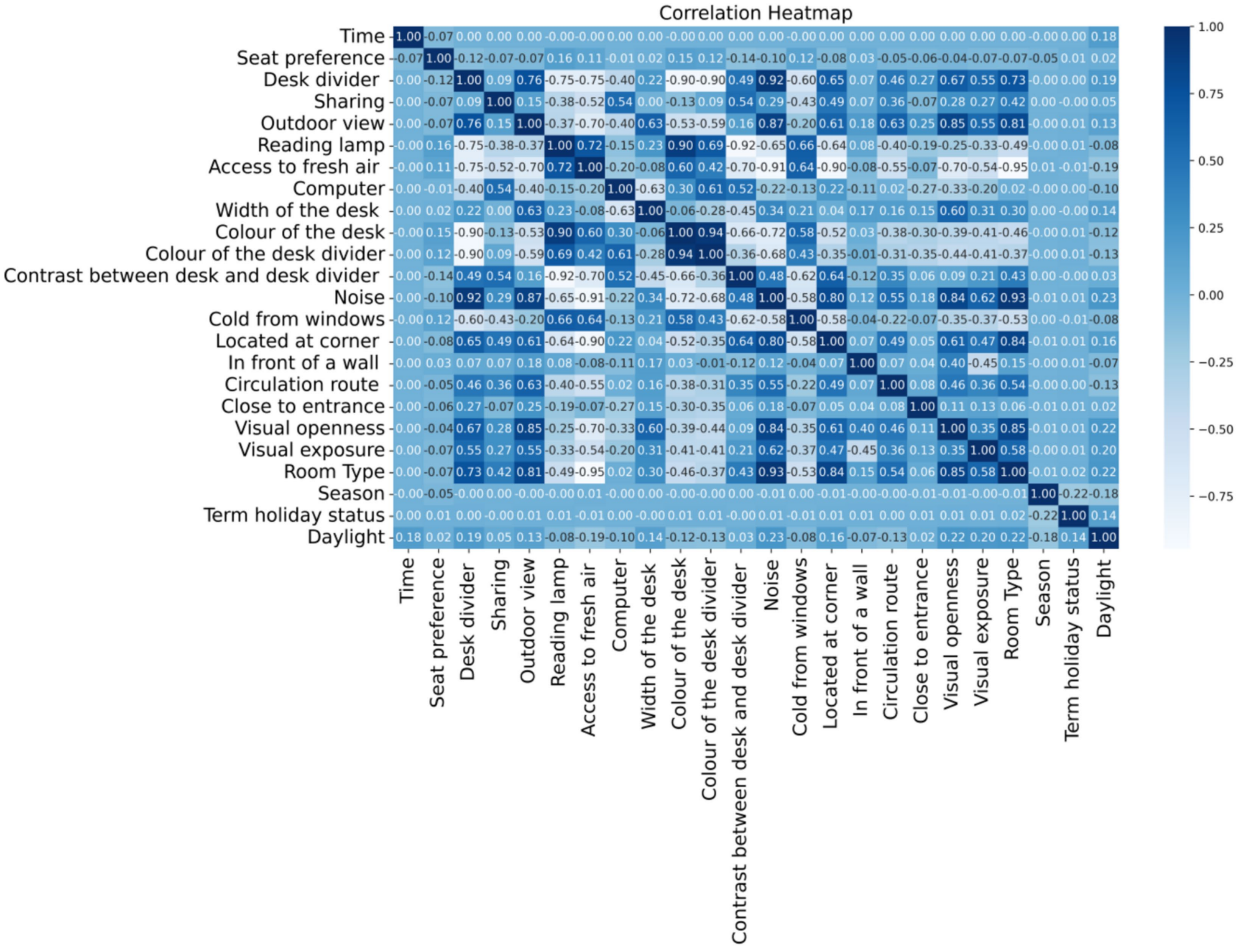
Percentage of samples labeled as “yes” (seat occupied) and “no”(seat not occupied) for each distinct feature value.

The correlations among the features based on Pearson correlation coefficients are visualized in the heatmap presented in Figure 7. Darker blue indicates a stronger correlation, whereas light blue or white values show little or no correlation. Given that the primary focus of this study is the seat preference attribute, special attention was paid to its pairwise correlations with other features. While none of the features showed a strong linear relationship (i.e., no correlation coefficient exceeded 0.5 in magnitude), several features displayed moderate positive or negative associations with seat preference as follows; “Reading lamp,” “color of the desk,” “thermal proximity to windows,” and “color of the desk divider” are the leading ones that have slight positive correlations with seat preference. Availability of a reading lamp is the most positively correlated factor that students slightly prefer seats with better lighting. Desk color may have an aesthetic comfort influence. The positive correlation in “thermal proximity to windows” seems counterintuitive, but it might suggest that colder seats are sometimes preferred, possibly confounded by window views.

The color of the desk divider may subtly influence preference, potentially through visual comfort. On the other hand, “the contrast between the desk and the desk divider,” “the desk divider,” “noise,” and “located at the corner” have the most influence in terms of negative correlation. High contrast may be visually disruptive or uncomfortable. The desk divider may reduce openness or perceived comfort. As expected, noisier areas are less preferred. Seats in corners may feel isolated or constrained.

“The attributes ‘Term Holiday Status’, ‘Computer’, ‘Width of the Desk’, and ‘Daylight’ show negligible correlation with seat preference, suggesting they do not substantially impact seat selection behavior. In summary, no feature shows a strong correlation (≥ ± 0.5) with seat preference, which indicates that seat preference is likely influenced by a combination of many weak factors, rather than one dominant factor. Nonlinear models or feature interactions may capture more complex patterns.

#### 3.2.2 Mutual-information ranking

Pearson correlation captures only linear relationships. Because there is no obvious linear relationship between the features and the seat preference, selecting the most informative ones that significantly affect the target feature is essential before applying machine learning methods. For this purpose, a nonlinear method, namely Mutual Information (MI), was employed in this study. Unlike correlation (which measures linear relationships), MI can capture any kind of dependency; linear or nonlinear, monotonic or not, using a model-independent filter method that selects features based on their intrinsic properties, independent of any machine learning model (Vergara and Estévez, 2014). It explains how knowing a feature’s value reduces uncertainty about the target variable. Because it is model-independent, the process is fast without needing to train machine learning models to evaluate features.

MI is applied step by step as follows: (1) the joint probability distribution of the feature and the target is estimated, (2) MI is calculated using Equation 2 for each feature concerning the target, where p(x,y) denotes their joint probability, and p(x) and p(y) are the marginal probabilities of the feature *X* and target *Y*, respectively. (3) The features are ordered according to their MI scores, and (4) the top-k features, those with the highest MI scores, are selected as the most informative *k* features.


$$\mathrm{MI}(X,Y)=\sum_{x\in X}\sum_{y\in Y}p(x,y)\log(p(x,y)/(p(x)p(y)))$$ (2)

The machine learning methods were first integrated with a feature selection process using mutual information. Based on the feature subset that yielded the highest classification accuracy, a subsequent step of hyperparameter tuning was carried out to enhance model performance further. Through analysis utilizing both linear (Pearson correlation) and non-linear (Mutual Information) feature evaluation, it was shown that no single environmental or spatial attribute strongly predicts seat choice; instead, behavior is driven by a combination of modest effects. Mutual information interpretation reveals that two main thematic bundles dominate students’ preferences:

##### 3.2.2.1 Environmental-controllability bundle

These features collectively empower users with a degree of agency over their immediate study environment. *A personal lamp* allows students to fine-tune the lighting at their workspace according to their preferences and visual needs. This direct adjustment supports comfort during prolonged reading or computer work, especially under varying ambient light conditions. *Operable windows allow users to introduce fresh air into their immediate vicinity, offering real-time adjustments to* air quality and, to some extent, temperature. An operable window can also influence noise levels, as students may choose to open or close windows depending on outside activity. *Edge-zone temperature* refers to seats located at a space’s perimeter (edge-zones), which often experience different thermal conditions than the room’s core. Students in these areas may have more options to relocate, adjust window blinds, or take advantage of user-controlled features (such as radiator valves or nearby openings), thus indirectly enhancing their ability to regulate local comfort.

This bundle aligns with adaptive comfort theory, which holds that people experience greater satisfaction and well-being in indoor environments where they can control core comfort parameters such as lighting, air quality, and temperature. Rather than being passive recipients of uniform building conditions, users value and benefit from the ability to shape their own microclimate actively, improving physical comfort, psychological well-being, and perceived autonomy.

##### 3.2.2.2 Distraction-management bundle

These features collectively protect users from involuntary sensory input, creating a calmer and more focused study environment. *Low background speech* refers to seats in quieter library zones, away from areas with high levels of conversation or noise. This minimises cognitive load by reducing auditory distractions, supporting sustained concentration and mental performance. *Set-back from main circulation routes* describes desks distanced from busy aisles, corridors, or main walkways with frequent movement. By limiting exposure to foot traffic and unpredictable peripheral motion, these locations help reduce both acoustic and visual interruptions. *Low-contrast desk finishes* use visually subtle desk surfaces and partitions that avoid strong color contrasts or patterns. Such finishes create a visually calm workspace, helping to minimize visual “noise” and distractions in the user’s immediate field of view.

This bundle aligns with cognitive load theory and psychophysiological research, which emphasize that minimizing involuntary sensory disturbances, whether auditory or visual, is essential for supporting deep focus and working memory. Rather than being distracted by environmental “noise,” students can concentrate more fully, leading to improved learning outcomes and a more pleasant study experience.

These bundles consistently outweighed other factors and interacted to shape seat selection. While exact information contributions may vary, the consistent emergence of these bundles in MI ranking analysis points to their central role in governing students’ choices. This supports adaptive comfort and cognitive load theories, underscoring the importance of both control and calm for sustained, high-quality study. Reporting these as ‘dominant’ or ‘most influential’ bundles is therefore justified, even without precise percentages for each variable.

### 3.3 RQ2: machine learning performance in predicting seat utilization

#### 3.3.1 Model implementation and optimization

The experimental results were obtained by training models on both the complete feature set and the selected features. All machine learning models were optimized by tuning their hyperparameters individually for each fold during 5-fold cross-validation, ensuring fair and robust evaluation. For the DT model, two key hyperparameters were tuned: *min_samples_split*: the minimum number of samples required to split an internal node, which controls how deep the tree can grow and helps prevent overfitting, and *min_samples_leaf*: the minimum number of samples needed to be present in a leaf node, which affects how fine-grained the terminal decisions can be. The search space for *min_samples_split* ranged from 2 to 20, increasing in steps of 2, while *min_samples_leaf* was varied from 1 to 10. The Gini impurity metric was used to evaluate the quality of splits during tree construction, guiding the model in selecting the most informative features at each node.

For the LR model, several key hyperparameters were tuned to optimize performance. The *penalty* parameter, which determines the type of regularization applied to prevent overfitting, was explored using the options: “l1” (Lasso), “l2” (Ridge), “elasticnet” (a combination of L1 and L2), and “None” (no regularization). The *C* parameter, which controls the inverse of regularization strength, was searched over the values [0.01, 0.1, 1, 10]. Smaller values of *C* indicate stronger regularization, while larger values allow the model to fit the training data more closely. The *solver* parameter, which specifies the optimization algorithm used during model training, was tuned using the options: “liblinear,” “lbfgs,” and “saga,” each suited for different combinations of penalties and dataset characteristics. When the penalty was set to “elasticnet,” an additional parameter, *l1_ratio*, was introduced to determine the balance between L1 and L2 regularization. It was tuned using the values [0.25, 0.5, 0.75], where 0 represents pure L2 and 1 represents pure L1 regularization.

The learning rate (*η*) and the number of estimators (*n_estimators*) are critical hyperparameters that significantly influence the performance of gradient boosting models such as XGB, GBM, and LGBM. The learning rate controls the contribution of each individual tree to the final model. A lower learning rate leads to slower but more precise learning, requiring more trees to converge, while a larger learning rate speeds up training but may risk overshooting or overfitting. The number of estimators refers to the total number of boosting rounds or trees used to build the ensemble. More estimators can capture complex patterns, but may increase training time and overfitting if not regularized. In this study, the optimal learning rate was searched from the list as [0.01, 0.025, 0.05, 0.075, 0.1, 0.15, 0.2]. The number of estimators was tuned within the range 50 to 500 for XGB and LGBM, and 50 to 250 for GBM, increasing in steps of 50.

Another ensemble learning method, RF, was implemented by tuning two important hyperparameters: *max_depth* and *n_estimators*. The *max_depth* parameter defines the maximum depth of each decision tree in the forest, controlling how many splits each tree can make. A deeper tree can capture more complex patterns but may increase the risk of overfitting. The search range for *max_depth* included the values 5, 10, 15, 20, and *None,* where *None* allows the tree to grow without depth restriction until all leaves are pure or contain fewer than the minimum required samples. The *n_estimators* parameter specifies the number of trees in the forest. Increasing this value generally improves performance up to a point, but also increases computational cost. It was tuned using values from 50 to 250, in increments of 50, to find the optimal balance between accuracy and efficiency.

The final boosting method, CatBoost, was evaluated by tuning two key hyperparameters: *learning_rate* and *depth*. The *learning_rate* controls the step size at each iteration as the model updates its predictions. Lower values result in slower but more stable learning, potentially leading to better generalization. This parameter was optimized using the values [0.005, 0.01, 0.02, 0.03, 0.05]. The *depth* parameter determines the maximum depth of the trees used in the boosting process. Deeper trees can model more complex relationships but may also increase the risk of overfitting. The depth was tuned using integer values from 6 to 12, enabling the model to balance complexity and performance across various configurations.

#### 3.3.2 Performance evaluation

A set of standard performance metrics was employed to assess how effectively the applied machine learning models predicted students’ future seat preferences based on the criteria outlined in Table 1. Each metric is defined concerning the actual and predicted labels.

True Positive (TP) defines that the model correctly predicted “yes,” i.e., the student was predicted to occupy the seat, and actually did. True Negative (TN) expresses that the model correctly predicted “no,” i.e., the student was predicted not to occupy the seat, and indeed did not. False Positive (FP) points to an error case where the model incorrectly predicted “yes” that the student was predicted to occupy the seat, but did not. Another error case is False Negative (FN) in which the model incorrectly predicted “no,” i.e., the student was not to occupy the seat, but did.

The primary metric used here is classification accuracy, which quantifies the proportion of all correct predictions (both “yes” and “no”) among all predictions by measuring the overall correctness of the model in predicting seat occupancy as given in Equation 3:


Accuracy=(TP+TN)/(TP+FP+TN+FN) (3)

In addition to accuracy, the evaluation considered other important metrics to provide a more comprehensive assessment: recall, precision, F1-score, and Area Under the Curve (AUC). These metrics were computed for each model, and their weighted averages were reported to account for class imbalance.

The performance of each model was evaluated from multiple perspectives by analyzing these metrics collectively, ensuring a more robust and reliable comparison. Recall, also known as Sensitivity, is the proportion of correctly predicted occupied seats among all occupied seats. It gives the information that out of all students who took a seat, how many were correctly predicted as “yes” by using the formula in Equation 4:


Recall=TP/(TP+FN) (4)

The proportion of correctly predicted occupied seats among all seats the model predicted would be occupied is given by the Precision metric. It specifies that out of all the seats the model predicted would be occupied, how many actually were. The following equation (Equation 5) is applied in the background:


Precision=TP/(TP+FP) (5)

The common inference between precision and recall can be made using the F1-score, which is their harmonic average, by balancing FP and FN. It summarizes the model’s performance for predicting seat occupation in a single score as given in Equation 6:


$$F1-\text{Score}=2*\frac{\left(\text{Precision}*\text{Recall}\right)}{\left(\text{Precision}+\text{Recall}\right)}\;$$ (6)

Area Under the Curve (AUC) evaluates the model’s ability to distinguish between classes based on the predicted probabilities by computing the area under the Receiver Operating Characteristic (ROC) curve. A higher AUC indicates better class separability between the “yes” and “no” labels. An AUC closer to 1 means the model effectively distinguishes between labels.

Initial experiments were conducted using the default parameter settings of the aforementioned classifiers, without applying any feature selection method. Regarding classification accuracy, the Logistic Regression (LR) model yielded the lowest performance at 59.31%, followed by the Decision Tree (DT) with an accuracy of 65.72%. Compared to ensemble learning methods, these relatively low results are expected, as ensemble classifiers such as Random Forest, Gradient Boosting, and their variants are designed to improve predictive performance by combining multiple weak learners. This aggregation process enables them to reduce variance and bias more effectively than individual models like LR or DT, thereby producing more robust and accurate predictions in complex tasks such as seat preference classification. The tree-based ensemble learning model Random Forest (RF) improved slightly over the standalone Decision Tree (DT), achieving a classification accuracy of 66.10% compared to DT’s 65.72%. However, significantly better results were obtained using boosting-based ensemble methods, outperforming both RF and Logistic Regression (LR). Among these, CatBoost demonstrated the highest performance, reaching an accuracy of 72.26%, indicating its effectiveness in capturing complex, non-linear relationships within the data. The other boosting models also showed competitive performance, with XGBoost (XGB) achieving 72.06%, LightGBM (LGBM) achieving 71.71%, and Gradient Boosting Machine (GBM) reaching 70.21%. These results highlight the advantage of boosting techniques in modeling nuanced patterns for predicting seat preference, especially compared to traditional classifiers.

In the next step, Mutual Information (MI) was employed as a feature selection technique to identify the most representative features for predicting seat preference. The order of the features was found from the most informative one to the least as follows: Sharing, Width of the desk, Noise, Time, Access to fresh air, Room Type, Contrast between desk and desk divider, Desk divider, Term holiday status, Color of the desk, Located at corner, Reading lamp, Outdoor view, Season, Cold from windows, Color of the desk divider, Visual openness, Close to entrance, Visual exposure, Circulation route, Daylight, In front of a wall, Computer. The order indicates how much each feature reduces uncertainty about the target (seat preference: “yes”/"no).

It can be inferred that certain environmental and spatial factors play a more decisive role in student seat selection. For instance, whether a student is sharing a desk, the physical width of the desk and the ambient noise level around the seat emerged as critical factors influencing their preference. This aligns with practical expectations. For example, students may avoid seats in noisy or crowded areas when seeking a quiet study environment or choose wider desks for increased personal space and comfort. Students preparing for exams or working on individual assignments may prefer seats with more privacy and fewer distractions. In such cases, shared desks can be less desirable, as they may introduce unwanted noise, reduced personal space, or interruptions.

On the other hand, students engaged in group work or collaborative study may deliberately choose shared desks. This variability highlights why the “sharing” attribute is highly informative, and it directly reflects social and functional preferences, which are key determinants in seat selection behavior. Conversely, features such as the presence of a computer at the desk, whether the desk is positioned in front of a wall, and the amount of daylight in the area showed minimal predictive contribution. It suggests that either these factors have a genuinely limited influence on seat preference or that their effects are already implicitly captured by other, more informative variables, rendering them somewhat redundant.

Identifying and utilizing only the most impactful features is crucial, as it allows the classifier to achieve comparable or even improved accuracy with fewer inputs. This reduces computational cost and training time and also helps to minimize overfitting, making the model more generalizable to new data. Therefore, each model was evaluated using progressively larger feature sets, where features were added one at a time based on their ranking according to the MI scores. The feature subset that yielded the highest performance was then selected as the optimal configuration for that particular model. As a result of the feature selection process, the number of selected features (out of 23 total) varied across models: 21 for Decision Tree (DT), 20 for Random Forest (RF), 22 for LightGBM (LGBM), 19 for Logistic Regression (LR), 22 for Gradient Boosting Machine (GBM), 22 for XGBoost (XGB), and 23 for CatBoost. Among them, CatBoost required the full feature set to achieve its highest accuracy, while Logistic Regression performed best with the most reduced subset, using only 19 features. Subsequently, all models were fine-tuned using the hyperparameter ranges described in Section 4.3, and their optimal parameter values were determined based on classification accuracy.

Table 2 shows the 5-fold cross-validation results of each classifier in terms of the metrics, namely accuracy (ACC), precision (PRE), recall (REC), F1-score (F1), and area under the curve (AUC). CatBoost outperforms all other models across all five metrics. This indicates that CatBoost is the most effective model for predicting seat preference in this context, likely due to its strong handling of categorical features and ability to model complex, nonlinear relationships. LGBM and XGB show very similar performance, both substantially outperforming simpler models like DT, LR, and RF. Their high AUC values suggest strong discriminative ability between the “yes” and “no” seat preference classes. GBM and RF provide moderate performance with scores around 71.2–71.3% accuracy, slightly below boosting-based approaches XGB and CatBoost. Their performance is relatively balanced but lacks the fine-tuning capabilities of more advanced gradient boosting variants. DT performs surprisingly well as a baseline. With an accuracy of 71.65%, DT outperforms both RF and GBM, though it is slightly less robust in terms of generalization (lower AUC than CatBoost and XGB). It may still be helpful in scenarios where interpretability is prioritized. Logistic Regression (LR) performs the worst with the lowest accuracy (59.58%) and AUC (61.06%). It struggles to capture the complexity of the feature relationships. This result confirms that the problem is not linearly separable, reinforcing the value of tree-based and ensemble models.

**Table 2:** Results of the applied methods in terms of different performance measures (%).

**Method**	**ACC**	**PRE**	**REC**	**F1**	**AUC**
DT	71,65	71,86	71,65	71,71	78,95
LR	59,58	59,16	59,58	57,87	61,06
RF	71,19	71,34	71,19	71,23	78,33
GBM	71,31	71,34	71,31	71,32	78,43
XGB	72,27	72,45	72,27	72,32	79,75
LGBM	72,30	72,52	72,30	72,36	79,85
CatBoost	72,51	72,69	72,51	72,56	80,11

Despite using strong ensemble models and tuning, performance seems to plateau around 72–73%. Here are possible reasons:

- Human behavior is complex and inconsistent, especially with preferences like seat selection. Students may choose seats based on unobserved or unrecorded factors, such as Personal mood, temporary distractions (e.g., availability of friends), habits, or randomness. These introduce label noise that no model can fully capture.
- Although 23 features are available, they may not fully reflect the real decision-making context. Features like desk color or room type may have limited predictive value, and higher-resolution behavioral or spatial data might be needed. Possible missing features: Real-time seat availability, past seat history, peer influence, course schedules, or workload.
- If features for “yes” and “no” preferences significantly overlap in value distributions, it is harder for any classifier to separate them cleanly. Even with good mutual information scores, features may still interact non-discriminatively.
- Tree-based boosting models like CatBoost and LGBM are already very expressive. If performance does not improve further, the model has likely learned all it can from the available data. Adding model complexity (deep learning, ensembles of ensembles, etc.) may not help if the signal-to-noise ratio is low.

### 3.4 RQ3: design-relevant insights from explainable AI

Since CatBoost was identified as the best-performing model and achieved its optimal results using the entire feature set without any prior feature elimination, a follow-up experiment was conducted within the scope of Explainable Artificial Intelligence (XAI). Specifically, SHAP (SHapley Additive exPlanations) values were analyzed to interpret the model’s behavior by quantifying the individual impact of each feature on the prediction outcomes (Lundberg and Lee, 2017). This approach enabled a deeper understanding of how and to what extent each input feature contributed to the model’s decisions.

**Figure 8:**
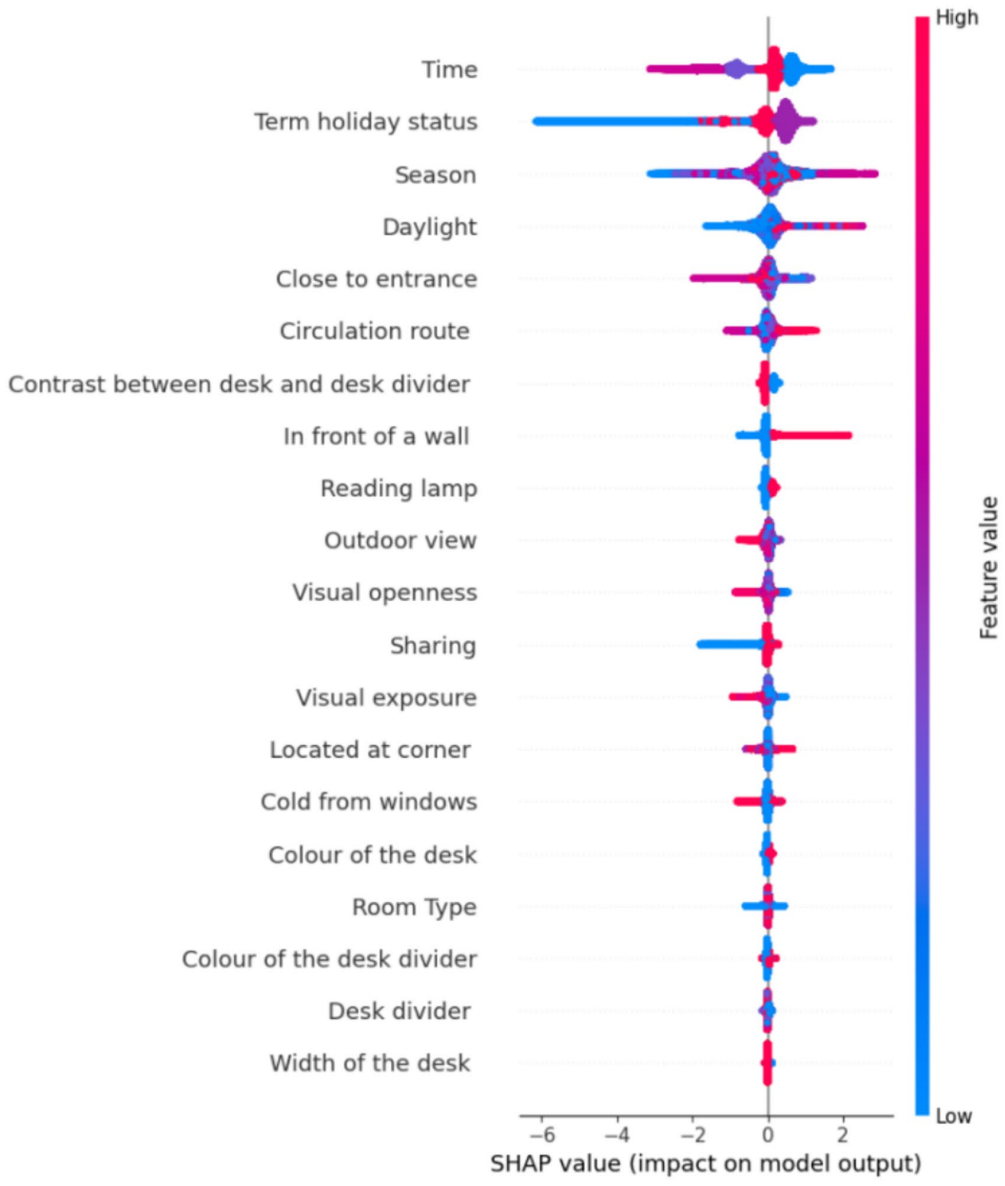
Impacts of the features on model output.

Figure 8 displays the resulting summary plot. It is designed in a way that the features are ranked from top to bottom on the y-axis according to their importance. The x-axis indicates how much a feature pushes the prediction away from the average toward a specific outcome, where a positive SHAP value pushes the prediction up, and a negative SHAP value pushes the prediction down. Another key factor is the color, where red points to high values, whereas blue points to low values of the features. Each dot represents a SHAP value for one sample.

- Top features (e.g., Time, Term holiday status, Season) have the most significant average impact on the model’s predictions.
- For Time, the blue and red points (Noon/Afternoon) mostly appear on the right side with positive SHAP values, and the purple and pink points (Morning/Evening) mostly appear on the left with negative SHAP values. Students are predicted to be more likely to choose seats in the afternoon or at noon. It is possible when students have breaks, return to study, or stay after classes. Morning and evening may reflect lower activity due to early lecture schedules, late arrivals, or a tendency to avoid early study hours.
- If Term holiday status is analyzed, SHAP values range both positive and negative, but blue dots (Holiday) mostly appear on the left side with negative SHAP values and pink dots (term time) cluster more on the right with positive SHAP values. This means that students are less likely to use seats during holidays, while the model is more likely to predict yes in the case of term time. It is reasonable that fewer students are on campus during official holidays, leading to lower seat preference. During the out-of-term, behavior is more variable and neutral.
- Season shows a moderate spread of SHAP values around zero. The blue dots (low values: Spring and Summer) are concentrated on the left side with negative SHAP values. The red dots (high values: Autumn and Winter) are more often on the right with positive SHAP values. The model is less likely to predict “yes” in Spring and Summer, whereas it is more likely to predict “yes” in Autumn and Winter.
- If daylight is analyzed, red (high daylight values) is mainly associated with negative SHAP values. Blue (low daylight) values cluster around zero or slightly positive. It is inferred that more daylight (red) slightly reduces the model’s likelihood of predicting “yes.” Students may avoid well-lit seats (e.g., to prevent glare or distractions). Less daylight (blue) has a neutral or slightly positive impact, suggesting dimmer areas might be more preferred, potentially for concentration or comfort.
- The “Close to entrance” feature is interpreted as blue points (very close to the entrance) are primarily on the right side with positive SHAP values, but red points (very far from the entrance) are primarily on the left side with negative SHAP values. Students prefer seats close to the entrance. Possibly for convenience, quicker access, or a sense of control over entering/exiting. Especially relevant for short visits, group work, or casual use. Seats far from the entrance are less preferred, potentially due to feeling isolated or being harder to reach or being less visible.
- Circulation route span both sides of the axis, but blue points (Seats that face away from a corridor (and thus movement flow) directly) are primarily on the left (negative SHAP values) whereas red points (Seats that have a distanced side-by-side directness with corridors) are more scattered or lean slightly more positive. Seats that face away from a corridor are less preferred and decrease the likelihood of being chosen. Seats with direct, straight-ahead visual contact with the main corridor have a neutral to slightly negative impact. Seats representing immediate side-by-side directness have a neutral effect, with neither consistently positive nor negative. Seats with a distanced side-by-side directness with corridors increase the chance of being chosen, so they are more preferred.
- Taking Contrast between the desk and the desk divider into consideration, blue dots (No contrast) tend to be on the right side of the plot by having positive SHAP values, and red dots (Contrast exists) appear more on the left side by having negative SHAP values. Students are more likely to choose seats where the desk and divider visually blend (no contrast). Strong contrast may be visually distracting, or feel more “separated” or institutional. A harmonious visual environment appears to support comfort and preference in seat selection.
- For the feature “In front of a wall,” red points (seat is in front of a wall) mostly appear on the left, while blue points (not in front of a wall) are more toward the right. Students are less likely to prefer seats that face a wall. Facing a wall may feel enclosed, visually restricted, or even claustrophobic. It might reduce access to natural light, external views, or a sense of space. Seats not in front of a wall provide a more open environment, which many students find more comfortable or stimulating.
- The blue points (not having reading lamp) in Reading lamp are on the left side, while the red points (having reading lamp) are on the right side, revealing that students strongly prefer seats with reading lamps, as indicated by consistently positive SHAP values for those cases. Providing better lighting enhances focus and is beneficial in low-light or cloudy conditions. Seats without reading lamps tend to be avoided, suggesting task lighting is a significant factor in seat selection behavior.
- In the case of outdoor view, blue dots (no outdoor view) appear mostly on the right side, and red dots (building view) appear more on the left side. According to this, students are more likely to prefer seats with no outdoor view. This may be due to fewer distractions, no windows, no movement, and no visual interruptions. Building views seem to impact preference negatively, possibly because they offer little aesthetic or daylight benefit. They may feel boxed in or less psychologically open than a wall with no view at all. While a partially outdoor view moderately increases the chance of seat selection, sky view negatively affects the case. The complete outdoor view shows no strong positive or negative influence, suggesting neutrality.
- Visual Openness in the library refers to the degree of visibility and exposure a student experiences while seated. Blue dots (low values: 0–1, i.e., very narrow to narrow) are on the right. This means that seats with low visual openness (narrow, confined spaces) tend to increase seat preference. Users may prefer such seats for privacy or reduced distractions. Red dots (high values: 4–8, medium to maximum openness) are on the left (negative SHAP values). This indicates that seats with high visual openness (very open spaces) decrease seat preference. Users may avoid these due to feelings of exposure or lack of privacy.
- The feature “Sharing” reflects whether a desk is shared and with how many people. Blue dots (no sharing) tend to be on the left, and red dots (shared with two people) tend to lean slightly to the right. A private, unshared desk decreases the preference for that seat (negative impact on the prediction). This could indicate that users prefer a more social or collaborative environment, even in a library. A seat shared with two other people slightly increases the preference for that seat by preferring a more socially engaging environment, such as study groups. Sharing with one person has a neutral effect on seat preference. It may not significantly influence user preference either way.
- Visual exposure represents the number of students who can potentially see a user’s computer screen at a workspace. Low Values (0–2 Students, Blue Dots) in the summary plot are primarily on the right. This indicates that seats with low visual exposure (very few people can see the screen) increase seat preference. Users likely prefer such seats for privacy and concentration. Medium Values (3–6 Students, Purple Dots) are closer to zero, suggesting a neutral impact. High Values (7–12 Students, Red Dots) are mostly on the left (negative SHAP values). This indicates that seats with high visual exposure (many people can see the screen) decrease seat preference. Users may avoid these seats to protect their privacy or minimize distractions.
- For the feature “Located at corner,” blue dots are spread around zero, indicating that when the corner status is not applicable, it has a neutral impact on seat preference. Red dots are slightly shifted to the right (positive SHAP values). This suggests that corner seats tend to positively impact preference, making them more likely to be chosen by students. They offer a sense of privacy and reduced distractions, which many library users may prefer for focused study. Purple dots (located not at the corner) slightly lean to the left (negative SHAP values), indicating that not being in a corner slightly decreases seat preference.
- Cold from windows refers to whether a desk is exposed to uncomfortable thermal conditions, particularly cold air near windows. Students appear indifferent to whether a desk is near a cold window. Possible explanations are as follows. Temperature variation near windows may not be severe or consistently uncomfortable. Other factors (e.g., lighting, privacy, desk size) might outweigh thermal concerns. The model did not learn a strong association between this feature and seat preference.
- SHAP values for the Color of the desk appear moderately close to zero. Blue points (White desks) tend to appear in the middle, giving a neutral effect, and red points (Wood desks) appear slightly more on the right. Students show a slight preference for wood-colored desks over white ones. Possible reasons are: Wood may be perceived as more natural or cosy by giving warmth and comfort. White desks may reflect more light, which could be visually distracting in bright conditions. The overall effect is small, meaning desk color alone does not drive strong decisions.
- Room Type has a neutral effect on seat preference. The SHAP values are clustered around zero. Blue to red points are evenly spread, with no particular value dominating. Students do not show a strong, consistent preference or aversion to any specific room type (small, main, hot desk space, skylight). This suggests that other physical or environmental features of a seat may be more influential in guiding their decisions.
- There is no dominant color trend aligning with strong SHAP values for the Color of the desk divider. The visual material or color of the desk divider (wood/dark) appears to have little influence on students’ seating decisions. Aesthetic features like color may be secondary to factors like light, comfort, or privacy.
- A similar issue is observed for the Desk divider, where no strong directional effect is visible. While different divider types exist, the model does not strongly favor or disfavor any single configuration.
- SHAP values for “Width of the desk” are clustered tightly around zero. There is no strong skew to the left or right. Both blue (narrow desks) and red (wide desks) points are distributed fairly symmetrically. It suggests that the width of the desk has a neutral or minimal influence on the model’s prediction.

### 3.5 Key findings

RQ1: Which spatial and environmental attributes most strongly influence long-term seat occupancy in an academic library?

The analysis revealed that students’ long-term seat occupancy is most strongly influenced by two interrelated bundles of factors: Environmental-controllability features, including the presence of a personal reading lamp, access to operable windows (fresh air), and seats located in edge zones with distinct thermal conditions, were consistently associated with higher seat occupancy. These attributes grant users greater agency over their immediate environment, supporting adaptive comfort and autonomy. Distraction-management features, such as low ambient noise, distance from main circulation routes, and minimal visual contrast between desk and divider, played a critical role, with students favoring locations that reduce auditory and visual distractions. Other positive predictors included proximity to entrances, corner seating (for privacy), and slight preferences for wood-toned desks. In contrast, factors like high visual exposure (many people can see the user’s screen), seats facing a wall, strong contrast between the desk and divider, and high noise levels were negatively associated with seat selection. Variables such as daylight availability, room type, computer presence, and desk width showed minimal influence on occupancy.

RQ2: How accurately can advanced machine learning models predict seat utilization, and how do their performances compare to baseline classification algorithms?

Advanced machine learning models, especially gradient boosting approaches such as CatBoost, XGBoost, and LightGBM, achieved substantial improvements in predictive performance compared to baseline models. CatBoost, the best-performing model, reached a classification accuracy of 72.51%, with comparable precision, recall, and F1-scores. These ensemble methods significantly outperformed simpler models such as logistic regression (59.58% accuracy) and standalone decision trees (71.65% accuracy). The results indicate that while advanced models can capture complex, nonlinear relationships in seat selection, the inherent unpredictability of human behavior and unmeasured contextual factors limit maximum achievable accuracy to around 72–73%.

RQ3: What design-relevant insights can be derived from explainable AI techniques (e.g., SHAP) to inform more user-responsive library seating layouts?

Explainable AI techniques, notably SHAP (Shapley Additive exPlanations), provided granular insight into the relative influence of each spatial and environmental feature on seat selection predictions. The SHAP analysis confirmed the primacy of features that enable environmental control and reduce distraction: seats with adjustable lighting, access to fresh air, and positions away from busy corridors were consistently favored. Visual privacy (lower visual exposure), subtle desk/divider contrasts, and avoiding seats facing walls further emerged as design priorities. Importantly, SHAP interpretation also revealed that some commonly assumed drivers of preference, such as daylight availability or desk size, are less influential than previously thought. This highlights the need for library layouts emphasizing user agency (control over comfort conditions), sensory calm, and visual harmony over traditional “one-size-fits-all” solutions. Overall, explainable AI improved model transparency and translated predictive results into actionable, evidence-based design recommendations for academic libraries.

## 4 Discussion

### 4.1 Interpretation of results

The findings of this study confirm that no single environmental or spatial attribute dominates seat choice in academic libraries; instead, seat selection is shaped by the interplay of multiple modestly influential factors. The low Pearson correlation coefficients observed for all variables (|*r*| < 0.20) support prior behavioral research on the “small-effect world” of environmental preferences (Lee Rodgers and Wander, 1988). Only the presence of a personal reading lamp and access to an operable window showed any notable, though still modest, positive associations with seat preference, in line with evidence that occupant-controlled lighting and fresh-air access can enhance perceived comfort and concentration (Veitch and Newsham, 2000; Brager and De Dear, 1998; Kim and de Dear, 2013).

Conversely, the most substantial negative influences were audible noise, adjacent to main circulation routes, and strong contrast between the desk and the divider. These reinforce extensive evidence that both acoustic and visual distractions hinder cognitive performance in shared environments (Hongisto, 2005; Haapakangas et al., 2018; Gordon-Hickey and Lemley, 2012).

Deeper analysis using mutual information (MI) revealed that the most informative predictors group into two main conceptual bundles: environmental controllability (control over lighting, access to fresh air, and edge-zone thermal conditions) and distraction management (reducing exposure to unwanted noise or visual distractions). However, the boundaries between these bundles are not always rigid; for instance, operable windows can offer users fresh air and help mitigate distractions, such as stuffiness or unwanted odors. Recognizing such overlap highlights the importance of holistic spatial strategies that address multiple user needs simultaneously.

In contrast to common design assumptions, daylight availability was not a primary determinant of seat preference in this study. Both correlation and feature importance analyses indicated only a weak influence of daylight on seat selection. SHAP analysis suggested that higher daylight levels might be less preferred, possibly due to glare or distraction; however, our data did not support a detailed analysis of conditional or interaction effects. This aligns with research cautioning that daylight’s benefits are highly context-dependent, being greatest when glare and thermal discomfort are minimized (Boyce, 2014). From a practical standpoint, this suggests that while daylight can enhance spatial quality, its design should prioritize glare control and complement other comfort and distraction-mitigating features, rather than serve as the sole or primary focus in library planning.

Advanced machine learning, particularly CatBoost, confirmed that seat preference is best predicted by the non-linear combination of comfort-enhancing and distraction-reducing features, rather than by any single factor. SHAP value interpretation illustrated that seat preference is maximized when multiple positive features coincide, but is strongly diminished by the presence of unwanted noise, even if other features are favorable (Lundberg and Lee, 2017; Hongisto, 2005)Despite advanced modeling, the plateau in predictive accuracy around 72% is consistent with reviews describing a behavioral “noise floor” in occupant behavior research (Yan et al., 2015; Dutta and Roy, 2022), highlighting the inherently complex and context-dependent nature of human choice. Importantly, the patterns identified here may not apply uniformly to all users. For example, preferences could differ by academic discipline, study purpose (individual vs. group), or even time of day, as suggested by recent research on the diversity of library user needs (Applegate, 2009). While the current study’s dataset did not allow for detailed subgroup analysis, future work could profitably examine how demographic, academic, or psychological differences shape seat selection and environmental preferences.

Finally, temporal segmentation of the data showed that these behavioral patterns were robust across term weeks, examination periods, and academic breaks, echoing findings on the stability of occupant adaptation behaviors such as window opening, daylight seat choice, and thermostat adjustment (O’Brien and Gunay, 2014; Aries et al., 2015; Hong et al., 2017).

In summary, this study demonstrates that library seat selection arises from the joint effect of comfort-related and distraction-mitigating features, with commonly assumed drivers such as daylight playing only a minor and context-dependent role. The evidence supports and extends adaptive comfort and cognitive load theories in the library context, emphasizing the need for holistic, evidence-based, and user-responsive design strategies. Future research should consider layering in dynamic social variables (e.g., group size, collaboration intent), physiological measures of stress or engagement, and explicit analysis of user subgroups to further unravel the nuanced drivers of spatial behavior in learning environments.

### 4.2 Implications for library design

The findings of this study offer actionable guidance for academic library design and management, emphasizing the value of user-responsive and evidence-based strategies over generic, one-size-fits-all approaches. Results consistently show that students’ seating preferences are shaped not by isolated attributes, such as desk size or daylight alone, but by the combined effect of comfort-enhancing and distraction-reducing features. This understanding is crucial for both the creation of new library spaces and the refurbishment of existing ones.

First, prioritizing environmental controllability is essential. Providing students with individual task lighting (such as personal reading lamps) and access to operable windows for fresh air emerged as significant factors in seat choice. These features allow users to adjust their immediate environment according to personal comfort needs, supporting adaptive comfort and promoting autonomy (Brager and De Dear, 1998; O’Brien and Gunay, 2014). Library design should therefore include locally controllable lighting and opportunities for natural ventilation, especially in study zones intended for prolonged use.

Second, minimizing unwanted distractions, both acoustic and visual, is critical. Seats located away from busy corridors and with reduced exposure to noise were consistently preferred. The disruptive effect of intense color contrast between desks and dividers further highlights the value of visually harmonious finishes. Designers should consider zoning study areas to buffer quiet, individual study seats from high-traffic walkways and group workspaces, using sound-absorbing materials and muted or natural surface finishes to create a visually calm environment (Hongisto, 2005; Haapakangas et al., 2018; Gordon-Hickey and Lemley, 2012).

Daylight, while a valuable aspect of spatial quality, did not emerge as a primary determinant of seat choice in this study. Its positive effects were only evident when daylight was carefully managed to avoid glare and discomfort. Rather than relying on large windows or skylights alone, effective library design should integrate shading devices, glare control, and flexible seating arrangements that allow students to select preferred light conditions (Boyce, 2014). Daylight can be leveraged as an amenity, but should be balanced with user comfort and control.

Factors such as desk width and fixed computer availability were found to have minimal influence on seat preference, challenging longstanding assumptions in library planning. Rather than maximizing desk size or digital infrastructure at each seat, greater emphasis should be placed on flexibility, privacy, and user control over the immediate study environment.

The robustness of seat preferences across different times and academic calendar phases suggests that these recommendations are broadly applicable and not tied to specific seasons or usage patterns. Nevertheless, acknowledging user diversity remains essential. Libraries should offer a variety of seat types and environments, quiet vs. collaborative, open vs. enclosed, reflecting the varying needs of individuals, tasks, and groups (Applegate, 2009).

It is also important to note that even advanced machine learning models could not capture all aspects of user behavior, underscoring the ongoing influence of social dynamics, personal routines, and psychological factors. As a result, ongoing post-occupancy evaluation and engagement with users should be central to the library design process, ensuring that spaces remain adaptable and genuinely supportive of student well-being and academic success (O’Brien and Gunay, 2014). Where relevant, library planners may wish to consult established best practice guidelines, such as IES recommendations for lighting, ASHRAE for thermal comfort, or ISO for acoustics, as operational benchmarks; however, specific thresholds should be selected with care, as this study did not directly measure or validate compliance with these standards.

In summary, this study advocates for library environments that empower students to control their immediate surroundings, minimize sources of distraction, and offer flexibility and inclusivity. Such spaces are well-positioned to support the diverse and evolving needs of today’s learners and to remain resilient as educational practices and technologies continue to change.

### 4.3 Limitations and directions for future research

While this study offers valuable insights into the determinants of students’ seating preferences in academic libraries, several limitations should be acknowledged. Firstly, this research was conducted at a single site, the UCL Bartlett Library, within a specific cultural, climatic, and institutional context. Although the comprehensive dataset spans a full academic year, the findings may not be fully generalizable to libraries with different layouts, user populations, or environmental conditions. Moreover, comfort priorities can vary with climate, building typology, and disciplinary culture; thus, the relative importance of comfort and distraction factors reported here may differ in science libraries, 24-h learning commons, or buildings in other regions. Replication across multiple libraries, climates, and academic cultures, ideally over several academic years, will be essential to validate and generalize these findings for broader design guidance.

Another methodological constraint is the reliance on PIR sensor-based occupancy data, which allows for objective, high-resolution tracking of seat usage. It cannot distinguish between multiple users at shared tables, record sustained presence, or identify the type of activity or collaboration. The system records motion but not continuous presence, and while obvious false positives were filtered, some errors may remain. Privacy-compliant combinations of PIR, depth cameras, and Wi-Fi localization have achieved higher accuracy in recent studies and merit consideration once ethical approval is secured.

A further limitation is that seat choice was correlated with simulated daylight but not with continuous desk-level measurements of temperature, humidity, CO₂ concentration, or octave-band noise. Consequently, brief periods of glare, thermal discomfort, or acoustic peaks could not be matched to occupancy events. Incorporating low-cost data loggers that stream these physical variables in future work would close this gap and align with recent recommendations for multimodal occupant research.

Notably, the study was observational rather than experimental. In-semester manipulation of lighting, ventilation, or desk orientation was not feasible, as it would have disrupted normal operations and potentially breached research ethics protocols. As a result, the associations revealed by mutual-information ranking and machine learning models cannot entirely eliminate confounding variables. Semester-long, randomized interventions, such as adding task lamps to a subset of carrels or implementing acoustic treatments, would provide much more substantial causal evidence when feasible.

Data collection covered only one academic year; unusual events such as renovation work, pandemic restrictions, or extreme weather outside the observation window could alter seat-selection patterns. Multi-year monitoring would be valuable to test the stability of the identified preference patterns under such exogenous shocks.

Another significant limitation is that subjective outcomes, such as perceived comfort, well-being, productivity, or satisfaction, were not directly measured, nor were any demographic or user profile data collected. Occupancy traces indicate what seats are chosen but not why. As a result, the current model operates under a one-size-fits-all assumption, which may overlook important differences in seating behavior across user subgroups. Factors such as age, gender, task type, frequency of library use, or even academic discipline may influence tolerance to noise, preference for daylight, or need for privacy. Incorporating such heterogeneous behavioral insights in future research could significantly enhance the explanatory power, personalization, and real-world applicability of seat preference models.

Additionally, while the predictive models used in this study were relatively accurate, they reached a performance plateau around 72% accuracy. This suggests that some behavioral “noise” or unmeasured factors limit the predictability of seat choice. Exploring additional features, interaction effects, or alternative modeling methods, such as deep learning or agent-based simulation, may improve predictive power and deepen understanding of complex human-environment relationships.

This study did not directly assess the impact of specific design or operational interventions, such as installing new lighting, introducing acoustic measures, or deploying digital feedback systems. Controlled field experiments and post-occupancy evaluations of targeted changes would provide stronger causal evidence for the practical effectiveness of proposed design solutions.

In summary, these limitations do not undermine the central finding that personal control and reduced distraction jointly govern seat choice. Instead, they define the boundary conditions for this study and point to clear priorities for future research: continuous environmental sensing, ethically approved field experiments, mixed-method inquiry, and multi-site replication. Advancing along these lines will refine evidence-based guidance for creating academic library environments that are both comfortable and adaptable to users’ diverse and evolving needs.

## 5 Conclusion

This study offers one of the most comprehensive, data-driven examinations of students’ seating preferences in academic libraries, combining a year-long PIR sensor dataset, advanced machine learning, and explainable AI to uncover the underlying drivers of seat selection in a real-world educational setting. The analysis demonstrates that seat choice is not dictated by any single environmental or spatial attribute, but by the nuanced interplay of multiple factors that foster comfort, autonomy, and freedom from distraction.

Two key bundles, environmental controllability and distraction management, consistently emerged as the strongest predictors of seat preference. Features such as user-controlled lighting, access to fresh air, and reduced exposure to noise and movement enhance physical comfort and support psychological well-being and sustained academic engagement. In contrast, factors traditionally presumed important, such as daylight availability, desk size, or built-in computers, had negligible influence on real-world user behavior once the broader context of comfort and distraction was considered.

The application of advanced machine learning, particularly ensemble methods and explainable AI, revealed both the complexity and the inherent variability of user preferences. Even the most accurate models plateaued at around 72% accuracy, reflecting the persistent influence of contextual, social, and unmeasured factors on human behavior in learning environments. Importantly, explainable AI tools (such as SHAP) enabled the translation of these complex models into clear, actionable guidance for spatial design and management.

This research advances our understanding of how students interact with academic library spaces by moving beyond conventional design assumptions and employing robust, objective methodologies. The findings advocate for library environments that prioritize user control, minimize sources of distraction, and provide flexible, inclusive settings capable of adapting to the evolving needs of diverse learners. Furthermore, the study demonstrates the societal and institutional value of data-driven, student-centered design in promoting student well-being, learning outcomes, and engagement.

While this research is based in a single, design-focused institution, its methodological approach and core insights are relevant across various educational contexts. Replicating this protocol in diverse cultural, climatic, and institutional settings will be crucial to establishing the best global library design and operation practices. The limitations of this work, such as its observational design and lack of subjective or demographic data, also point the way for future research, which should integrate multimodal environmental sensing, qualitative user input, controlled interventions, and multi-site replication.

Ultimately, this study underscores that optimizing library seating and spatial experience is not merely a question of furniture layout or lighting provision, but of fostering environments where students can thrive academically, physically, and psychologically. As educational spaces and technologies evolve, libraries that combine advanced analytics with user-centered design principles will play a transformative role in supporting student success and institutional resilience in a rapidly changing world.

## Data Availability

The raw data supporting the conclusions of this article will be made available by the authors, without undue reservation.
