# Correction: From motion to meaning: understanding students' seating preferences in libraries through PIR-enabled machine learning and explainable AI

**DOI:** 10.3389/fpsyg.2025.1729654

**Published:** 2026-01-12

**Authors:** Gizem Izmir Tunahan, Goksu Tuysuzoglu, Hector Altamirano

**Affiliations:** 1 Department of Architecture, Dokuz Eylul University, Izmir, Türkiye; 2 Department of Computer Engineering, Dokuz Eylul University, Izmir, Türkiye; 3 UCL Institute for Environmental Design and Engineering, London, United Kingdom

**Keywords:** seat preference, occupancy monitoring, academic library, machine learning, explainable AI, spatial behavior, user comfort, PIR sensors

In the published article, the **Conflict of interest** statement was erroneously given as:

The authors declare that the research was conducted in the absence of any commercial or financial relationships that could be construed as a potential conflict of interest.

The correct conflict of interest statement is below:

The author(s) declared that that this work was conducted in the absence of any commercial or financial relationships that could be construed as a potential conflict of interest.

Author GI voluntarily disclosed a collaboration on a different article with reviewer ZND that started during the review of this article. GI at the time of the review was not aware that ZND was one of reviewers of the paper. This had no impact on the peer review process and the final decision.

The original version of this article has been updated.

